# Population genetic structure of *Pomacea canaliculata* in China based on the *COI* and *ITS1* genes

**DOI:** 10.1038/s41598-024-62554-6

**Published:** 2024-05-27

**Authors:** Ran Wei, Ya-Wen Chang, Hong-Fang Xie, Cheng-dong Wu, Deng-Rong Yuan, Wei-Rong Gong, Yu-Zhou Du

**Affiliations:** 1 College of Plant Protection, Yangzhou University, Yangzhou, 225009 China; 2 College of Bioscience and Biotechnology, Yangzhou University, Yangzhou, 225009 China; 3 Plant Protection and Quarantine Station of Nanjing City, Nanjing, 210029 Jiangsu Province China; 4 Pukou Agricultural Technology Extension Center of Nanjing City, Nanjing, 211800 China; 5 Plant Protection and Quarantine Station of Jiangsu Province, Nanjing, 210036 China

**Keywords:** *Pomacea canaliculata*, Genetic diversity, Genetic structure, MtDNA *COI*, rDNA *ITS1*, Evolution, Genetics, Molecular biology

## Abstract

Comprehending the phylogeography of invasive organisms enhances our insight into their distribution dynamics, which is instrumental for the development of effective prevention and management strategies. In China, *Pomacea canaliculata* and *Pomacea maculata* are the two most widespread and damaging species of the non-native *Pomacea* spp.. Given this species’ rapid spread throughout country, it is urgent to investigate the genetic diversity and structure of its different geographic populations, a task undertaken in the current study using the *COI* and *ITS1* mitochondrial and ribosomal DNA genes, respectively. The result of this study, based on a nationwide systematic survey, a collection of *Pomacea* spp., and the identification of cryptic species, showed that there is a degree of genetic diversity and differentiation in *P. canaliculata*, and that all of its variations are mainly due to differences between individuals within different geographical populations. Indeed, this species contains multiple haplotypes, but none of them form a systematic geographical population structure. Furthermore, the *COI* gene exhibits higher genetic diversity than the *ITS1* gene. Our study further clarifies the invasive pathways and dispersal patterns of *P. canaliculata* in China to provide a theoretical basis.

## Introduction

Despite ongoing efforts to understand the factors that influence the evolution of species, it is widely recognized that speciation is a complex process involving mutation, gene flow, natural selection, and genetic drift^1^. Natural selection is often considered the primary force shaping genetic diversity in species^2–4^, while neutral theories suggest that evolution is not natural selection, but rather random genetic drift^5^. In the last three decades, many authors have considered the neutral theory to be outdated^6^, but recent studies have demonstrated that short tandem repeat sequences (STRs) can bind transcription factor DNA-binding domains^7^. A new theory-the theory of maximum genetic diversity^8^ has also emerged, which retains the advantages of the neutral theory. Therefore, the present form of the neutral theory is incomplete and, despite its limited applicability, likely to remain an integral part of the exploration of molecular evolution^5^. The comparison of DNA sequences has become a valuable tool in studying the evolutionary forces at play^9^. For pests, insights into how phylogeography and populations’ evolutionary history patterns are shaped and their major associated evolutionary mechanisms are still lacking, but this is important for sustainable management and ecology and evolution studies^10–12^.

Mitochondrial DNA cytochrome c oxidase subunit I [*COI*] and ribosomal DNA Internal Transcribed Spacer I [*ITS1*] markers have been widely and successfully used to reveal the genetic structure and evolutionary biology of numerous agricultural pests, including pink rice borer^13^, *Spodoptera frugiperda*^14^, *Arctodiaptomus dorsalis*^15^, and *Aculops lycopersici*^16^. It is well known that ribosomal genes are commonly used in molecular phylogeny, kinship analysis, since *ITS* is under less natural selection pressure during the evolutionary process, more variation exists^17^, and the evolutionary rate of *ITS* fragments is 10 times higher than that of *18S* rDNA. Recent studies have found that *ITS1* is more efficient than *ITS2*, and its primers’ applicability are more general and widespread, making *ITS1* superior to *ITS2*. The evolutionary rate of mitochondrial genes is faster^18^ and is more effective in revealing haplotypes and population histories than that of ribosomal genes.

The *Pomacea* spp., freshwater gastropod snails native to the Amazon region of South America, has spread and become a worldwide pest, owing to its extreme adaptability, very wide host range, and lack of natural enemies^19,20^. The *Pomacea* spp. are a group consisting of several species, including *Pomacea canaliculata* and *Pomacea maculata*, the primary species introduced into China. These two snails have been frequently mistaken for one species because of their morphological similarities^21^, and, for a long time, *P. canaliculata* was considered to be the only invasive snail, meaning that the two were conflated in early literature reports^22^. Analyses of the *Pomacea* spp. based on mitochondrial genes appeared in 2011, followed by the molecular identification of the *Pomacea* spp. using *COI*^23,24^, and genetic differentiation analyses of different populations have gradually increased in recent years^25–29^, with *COI* also being widely used in population history dynamics studies^30–32^. However, an analysis of the *Pomacea* spp. based on ribosomal genes has not been reported. Therefore, it is extremely necessary to detect the geographical distribution of the genetic structure of *P. canaliculata* from the perspective of various populations based on the two genes.

In China, however, knowledge of the phylogeography and evolutionary biology of *Pomacea* spp. is limited compared to other pests. In this study, we explored the invasive pathway and dispersal patterns of *P. canaliculata* through an integrated phylogeographic approach. We collected a large number of *P. canaliculata* specimens from almost all the damaged areas in China (20 geographic populations in total) and analyzed genetic data with mitochondrial and ribosomal DNA *COI* and *ITS1*, respectively, as deciphering the phylogeography and population evolutionary history can facilitate the development of effective pest suppression protocols and sustainable management strategies^33,34^. The primary objectives of this study were to: (a) evaluate the genetic diversity of *P. canaliculata*; (b) investigate the evolutionary history of population in China.

## Materials and methods

### Sample collection and DNA extraction

In 2021–2022, we collected both snails and egg of *Pomacea* spp. from rivers, ponds, and public farmland at 20 different sites across China, almost covering all the regions. The total DNA from the evenly sized, collected adult snails was purified using the AxyPrep Genomic DNA Kit (Suzhou, China).

### The mitochondrial and ribosomal gene sequencing

**Figure 1:**
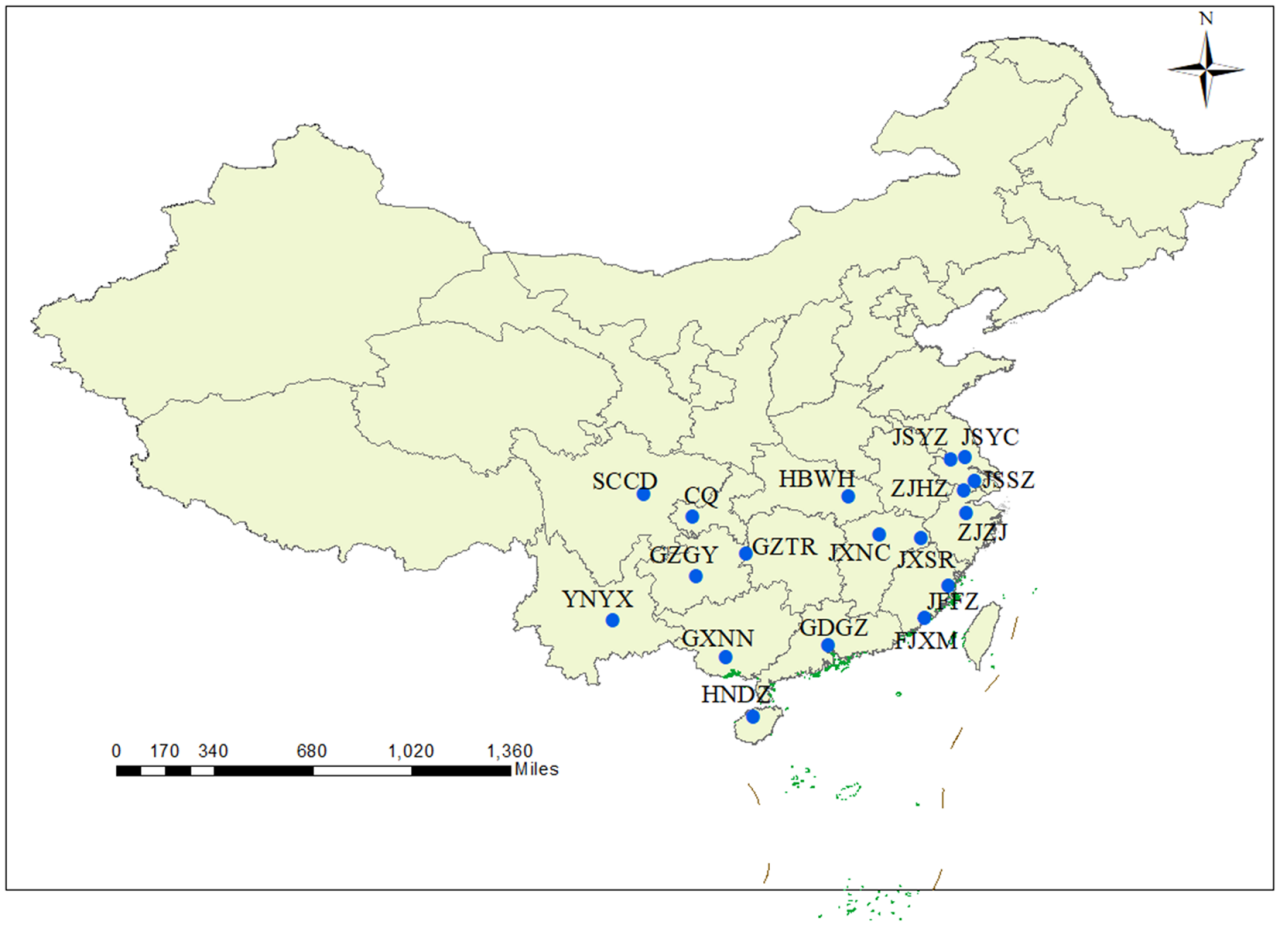
Sampling locations of 18 *P. canaliculata* populations in China: map created using Arcgis platform. Abbreviations described in Table 1.

The primers for COI and ITS1 were designed using Primer5. *COI* was amplified using Primers(F) (5’AGAGTGGTGCTGGAACTGGATGAA3’ and 5’CCGGTGCTCTATTAGGGGATGATCAA3’) and Reverse(R) (5’GCTAATATAGCATAAATTATTCCTAAAGTACC3’). The *ITS1* region was amplified using primers F (5’CGGATTGGTCTCGGCCCGCCCTTCA3’) and R (5’ATCCACCGCCTAAAGTTGTT3’) also used for the sequencing reactions. For the polymerase chain reactions (PCRs), we used 2μL DNA in the reaction mixture containing 12.5μL Taq Master Mix, 1μL of each primer, and 8.5μL ddH_2_O. The PCR temperature profile was the following: 94℃ for 3min, 94℃ for 1min, 55℃ for 45s, 72℃ for 1min, 35 cycles, and 72℃ for 10min. An electrophoretic analysis of the PCR fragments was performed on 1% agarose gel. The resulting PCR products were purified using a Gel DNA Extraction Kit (Vazyme) and sequenced with an ABI 3730XL DNA sequencer (Sangon Biotech, Shanghai). Using the National Center for Biotechnology Information (NCBI)-Blast (http://www.ncbi.nlm.nih.gov/BLAST) based on the *COI* gene against homologous sequences in GenBank, the highest sequence homology (> 97%) was identified for *P. canaliclata*. The distribution of *P. canaliculata* was screened, with 6 uniformly sized adult snails selected at each locus for subsequent studies; the locations are shown in Fig. 1 and Table 1. Sequences of 18 locations were submitted to the NCBI (GenBank accession nos. PP461874-PP461981 and PP448193-PP448300).

**Table 1:** Sample collecting information of *P. canaliculata* in this study.

Number	Collection locations	Code	Collecting date (month-day)	Latitude (°) longitude (°)	N
1	Hainan, Danzhou	HNDZ	Oct-12	109.58E/19.52N	6
2	Jiangsu, Suzhou	JSSZ	Mar-25	120.62E/31.32N	6
3	Jiangsu, Yangzhou	JSYZ	May-9	119.42E/32.39N	6
4	Jiangsu, Yancheng	JSYC	Sep-15	120.19E/32.51N	6
5	Yunnan, Yuxi	YNYX	Oct-14	102.54E/24.35N	6
6	Guangdong, Guangzhou	DGDZ	Oct-26	113.31E/23.12N	6
7	Zhejiang, Huzhou	ZJHZ	Oct-10	120.10E/29.71N	6
8	Zhejiang, Zhuji	ZJZJ	Oct-9	120.24E/29.71N	6
9	Chongqing	CQ	Oct-8	106.53E/29.54N	6
10	Hubei, Wuhan	HBWH	Nov-16	114.32E/30.58N	6
11	Sichuan, Chengdu	SCCD	Oct-13	104.07E/30.68N	6
12	Jiangxi, Nanchang	JXNC	Oct-24	115.89E/28.68N	6
13	Jiangxi, Shangrao	JXSR	Oct-14	117.97E/28.44N	6
14	Fujian, Fuzhou	FJFZ	Oct-23	119.31E/26.08N	6
15	Fujian, Xiamen	FJXM	Oct-27	118.11E/24.49N	6
16	Guizhou, Tongren	GZTR	Aug-13	109.19E/27.72N	6
17	Guizhou, Guiyang	GZGY	Oct-10	106.71E/26.58N	6
18	Guangxi, Nanning	GXNN	Oct-17	108.19E/22.49N	6

N, the number of mtDNA *COI* and rDNA *ITS1* sequences used.

### Genetic diversity analysis

We utilized CLUSTAL X 1.83^35^ to manually align the sequences after removing any redundant ones. Sequences were calculated for parameters such as parsimony informative sites (Pi), single mutation sites(S), variant sites(V), GC content of the sequences, reversion values(R) of the sequences, and distances within and among groups using MEGA X^36,37^. Genetic diversity was analyzed, including parameters such as the number of polymorphic sites(S), the total number of mutations(η)^38^, the number of haplotypes(H), nucleotide diversity (π), haplotype diversity (Hd)^39^, and the average number of nucleotide differences (K)^40^ using DNASP v. 5.0^41^.

### Population genetic structure analyses

We applied multiple complementary approaches to explore the population genetic structure of *P. canaliculata* based on both mtDNA and rDNA datasets.

Using the Bayesian Markov Chain Monte Carlo (MCMC) model in the STRUCTURE v2.3.3 software^42,43^. We performed this analysis with K ranging from 2 to 10, a burn-in of 10,000, and a run length of 100,000, each K-value obtained with 10 independent runs. The statistic “△K”, indicating the most likely number of subpopulations, was calculated following the Evanno method^44^, and then the Q matrix under the highest △K-value was obtained. Next, to visualize the genetic divergence between populations, a pairwise distance matrix derived from the *COI* and *ITS1* genes Nei’s genetic distance for all samples was calculated, and a neighbor-joining(NJ) tree was constructed using TASSEL v5.0, which was used to analyze kinship^45^. In addition, we utilized principal component analysis (PCA) in the R environment as a complementary method to identify the genetic structure of *P. canaliculata*.

F-statistics value (Fst; differentiation index), gene flow (Nm), and exact tests between the populations were calculated based on both mtDNA and rNDA datasets using the Arlequin v. 3.5 software^46^. and an analysis of molecular variance (AMOVA).

### Haplotype relationship analysis

We constructed split networks to reveal relationships among mitochondrial haplotypes using NETWORK v. 10.2.0.0^47,48^and performed a neighbour-net analysis under a Kimura-2-parameter (K2P) distance model after 1000 bootstraps.

### Demographic history analysis

The demographic history of *P. canaliculata* populations in China was studied using mismatch distributions data in DNASP v. 5.0^41^. Neutrality tests based on Tajima's D and Fu's Fs parameters were performed, with all the parameters evaluated based on 1000 bootstrap replicates.

## Result

### High level of genetic diversity

**Figure 2:**
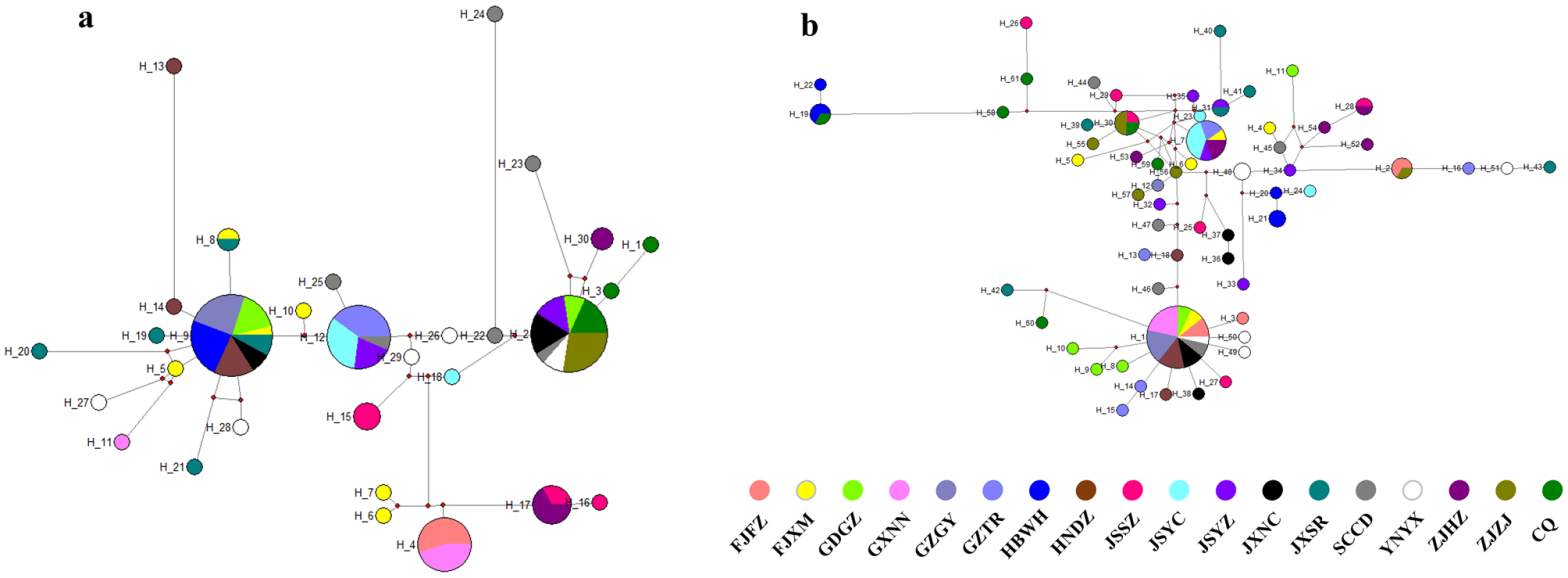
Haplotype-based network graphs of *P. canaliculata*. (**a**) based on mtDNA *COI* gene. (**b**) based on rDNA *ITS1* gene.

There were 445 base pairs in the mtDNA *COI* sequences, with 232 bp and 114 bp being variable and parsimony informative, respectively. For the *COI* genes, 30 haplotypes were identified (Supplementary Table S1 online), with 22 distinct haplotypes only present in one individual, the remaining 8 haplotypes present in at least two or more individuals, and Hap9 being the most prevalent haplotype in all populations. The majority of the mutation sites were found at position 1 (140, 136, and 135 at position 1, 2, 3), according to our analysis of the polymorphisms at each of the codon's positions (1, 2, or 3) (Supplementary Table S3 online). There were 118 single-ton variable sites. In addition, the rDNA *ITS1* sequences were 469 base pairs, with 89 bp and 56 bp being, respectively, variable being parsimony informative. There were 61 haplotypes found for the *ITS1* genes (Supplementary Table S2 online), of which 52 were distinct haplotypes found in just one individual, while the other 9 haplotypes were found in at least two or more individuals. Hap1 was the most common and ubiquitous haplotype across the populations. The majority of the mutation sites were found at position 3 (146, 144 and 147 at position 1, 2, 3), according to our analysis of the polymorphisms at each of the codon's positions (Supplementary Table S4 online); 33 of these were singleton variables. The genetic variance in the eighteen *P. canaliculata* populations in China varied, with the majority showing significant levels of variation. For the *COI* and *ITS1* genes independently, we created haplotype-based network graphs where each node corresponded to a haplotype (Fig. 2).

In terms of *COI*, the average haplotype diversity (Hd) across all the populations was 0.877, with an average nucleotide diversity (π) of 0.04304 and a mean number of nucleotide differences (K) of 14.719 (Table 2). Regarding *ITS1*, the mean haplotype diversity (Hd) across all the populations was 0.924, with a mean nucleotide diversity (π) of 0.02336 and an average number of nucleotide differences (K) of 10.161 (Table 2). Additionally, an evaluation of the genetic diversity of *P. canaliculata* was conducted by excluding unique haplotypes (e.g., Hap3 and Hap5) exclusive to individual *P. canaliculata*. Upon the removal of these distinctive haplotypes, the average haplotype diversity (Hd) for all the populations in the *COI* gene dropped to 0.812. The mean nucleotide diversity (π) across all the populations was 0.03600, with an average number of nucleotide differences (K) of 14.938. Similarly, for the *ITS1* gene, the average haplotype diversity (Hd) among all the populations decreased to 0.717, the mean nucleotide diversity (π) was 0.02257, and the average count of nucleotide differences (K) was 9.910. These parameters collectively indicate a substantial level of genetic diversity within *P. canaliculata*.

**Table 2:** Parameters of genetic diversity of 18 *P. canaliculata* populations based on mtDNA and rDNA.

Population	*COI*						*ITS1*					
	h	S	η	Hd	π	K	h	S	η	Hd	π	K
FJFZ	4	6	7	0.800	0.00703	3.067	4	11	11	0.800	0.01228	5.467
FJXM	6	42	44	1.000	0.04793	20.800	5	27	30	0.933	0.03029	13.600
GDGZ	4	20	20	0.800	0.02440	10.467	5	15	15	0.933	0.01153	5.200
GXNN	6	51	54	1.000	0.04262	18.667	1	0	0	0.000	0.0000	0.000
GZGY	5	8	10	0.933	0.00852	3.733	2	12	12	0.333	0.00885	4.000
GZTR	6	4	6	1.000	0.00535	2.333	5	26	28	0.933	0.02956	13.067
HBWH	5	5	7	0.933	0.00624	2.733	4	30	30	0.867	0.03891	17.667
HNDZ	6	48	52	1.000	0.04144	18.067	3	10	10	0.600	0.00737	3.333
JSSZ	6	39	43	1.000	0.05413	23.600	6	33	33	1.000	0.03197	14.067
JSYC	3	13	13	0.733	0.01037	4.533	3	16	17	0.600	0.01292	5.800
JSYZ	4	19	21	0.867	0.02676	11.667	6	28	30	1.000	0.02708	12.133
JXNC	4	24	25	0.800	0.02809	12.333	4	21	21	0.800	0.02411	10.800
JXSR	5	72	83	0.933	0.06778	28.400	6	30	31	1.000	0.02812	12.400
SCCD	6	78	85	1.000	0.08527	30.867	5	21	21	0.933	0.01778	8.000
YNYX	6	54	61	1.000	0.05679	24.533	5	23	23	0.933	0.02262	10.067
ZJHZ	3	49	49	0.733	0.06059	25.933	5	22	22	0.933	0.02405	10.800
ZJZJ	4	5	6	0.867	0.00566	2.467	5	18	18	0.933	0.01719	7.600
CQ	6	26	30	1.000	0.02442	10.600	6	35	38	1.000	0.03454	15.267

Abbreviations of populations and the number of individuals per population are described in Table 1.

*S* the number of polymorphic sites, *η* the total number of mutations, *h* the number of haplotypes per population, *Hd* haplotype diversity, *K* average number of nucleotide differences, *π* nucleotide diversity.

### Demography history

We determined the population genetic structure of *P. canaliculata* based on both mtDNA and rDNA datasets.

**Figure 3:**
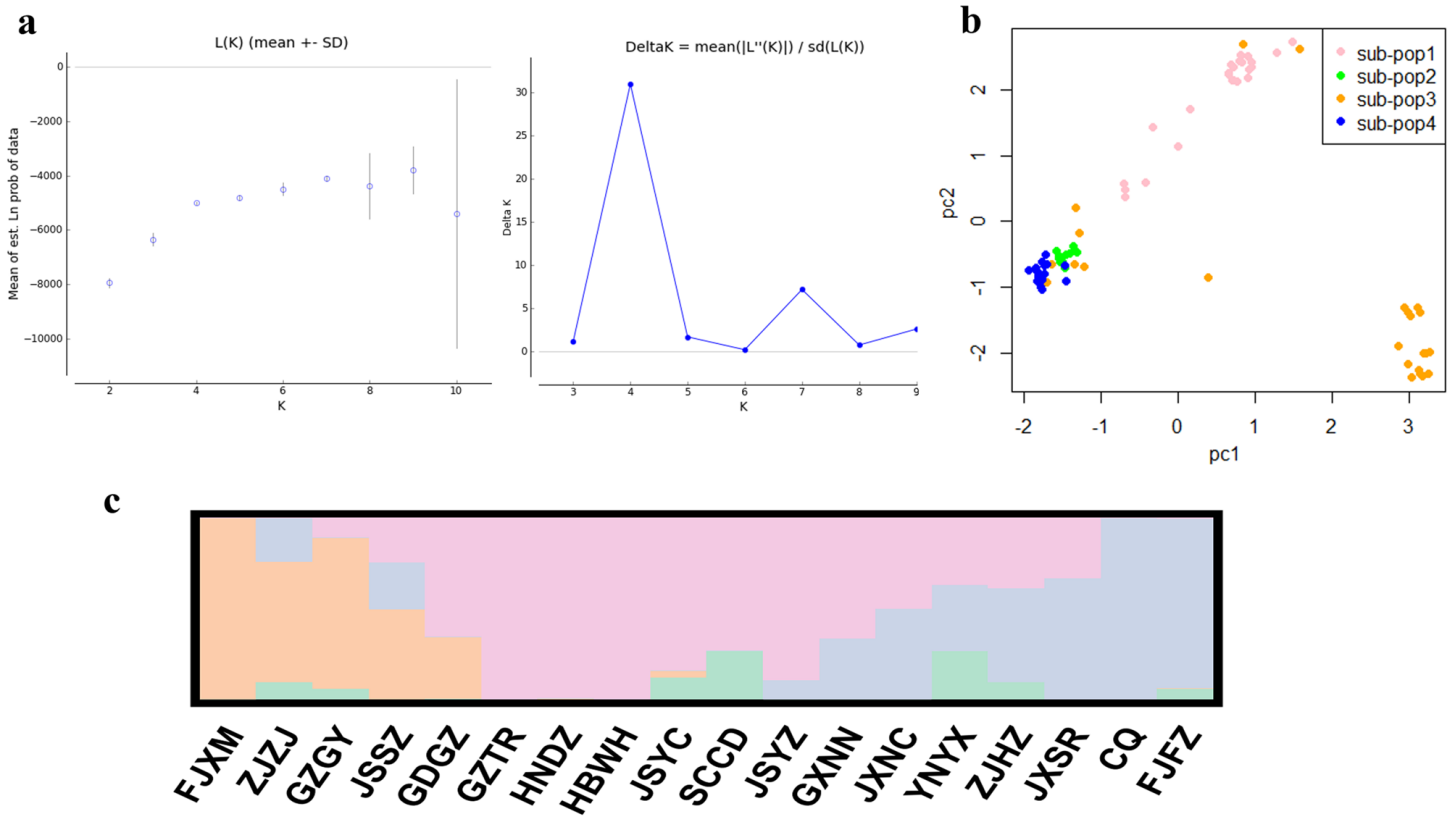
Four *P. canaliculata* subgroups in China based on mtDNA data analysis. **(a)** Graph showing the number of inferred clusters (K) using Evanno’s delta (K) method. **(b)** Principal components analysis plot for *P. canaliculata* populations based on mtDNA genotypes; two-dimensional scales are used to reveal population stratification. **(c)** Four *P. canaliculata* subgroups in China based on STRUCTURE analysis. Abbreviations of the populations are described in Table S1online.

**Figure 4:**
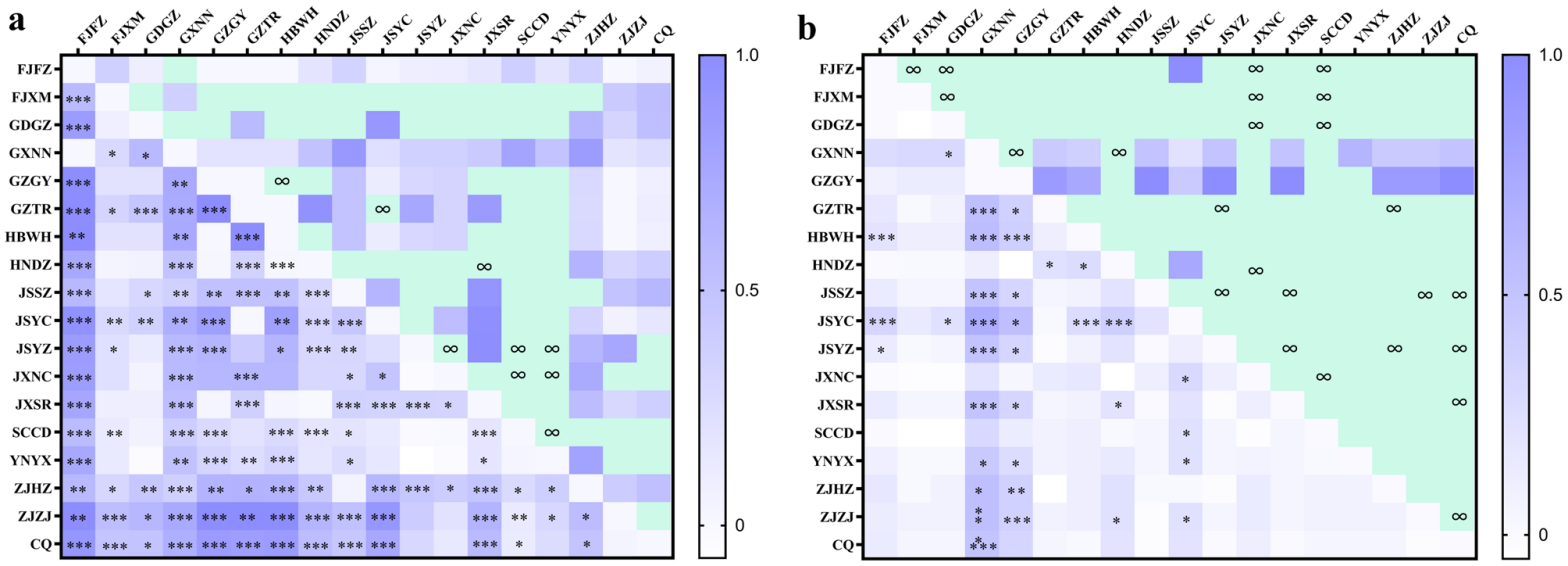
Heatmaps of genetic differentiation (Fst, lower left triangles) and gene flow (Nm, top right triangles) of different *P. canaliculata* populations in China using GraphPad Prism v8.0.2. **(a)** Analyzed using mtDNA *COI* gene. **(b)** Analyzed using rDNA *ITS1* gene. Note: *P < 0.05, **P < 0.01, ***P < 0.001, ∞ infinity, green squares indicate Nm > 1.

From the mtDNA data analyses, a Bayesian cluster analysis using STRUCTURE revealed an optimal value of K = 4 was the best fit for the 18 populations in China and a relatively high value of Evanno’s delta (K) (Fig. 3a). The F_ST_ value among all the geographical groups was 0.45296 (Table 3), indicating a high level of genetic differentiation among different geographic populations, alongside genetic exchange (Fig. 4a). The Exact test showed that 67.32% of the populations (Table S5 online) did not support the random mating group hypothesis (*P* < 0.05), suggesting that reproductive isolation has occurred between some of the geographic populations. As a result, a possible cryptic species of *P. canaliculata* was found based on the *COI* gene alone. The results of the NJ phylogenetic tree (Fig. S1a online) and PCA (Fig. 3b) were consistent with the population stratification obtained from the STRUCTURE software (Fig. 3c). Therefore, four more subgroups were identified (Fig. 3a): the first subgroup included the JSYZ, WBWH, JGZTR, GXNN, GDGZ, and HNDZ populations; the second subgroup contained the ZJHZ, JXNC, JXSR, FJFZ, and CQ populations; the third subgroup contained the JSYC, SCCD, and YNYX populations; and the fourth subgroup comprised the JSSZ, FJXM, GZGY, and ZJZJ populations.

**Table 3:** Analysis of molecular variance (AMOVA) of *COI* gene and *ITS1* gene for 108 individuals in 18 *P. canaliculata* populations.

	Source of variation		df	Sum of squares		Variance components		% of variation		*F*_*st*_	
mtDNA *COI*	Among populations		17	417.296		3.40565 Va		45.30		0.45296	
	Within populations		90	370.167		4.11296 Vb		54.70			
	Total		107	787.463		7.51861					
rDNA *ITS1*	Among populations		17	13.269		0.06311 Va		13.57		0.13573	
	Within populations		90	36.167		0.40185 Vb		86.43			
	Total		107	49.935		0.46496

**Figure 5:**
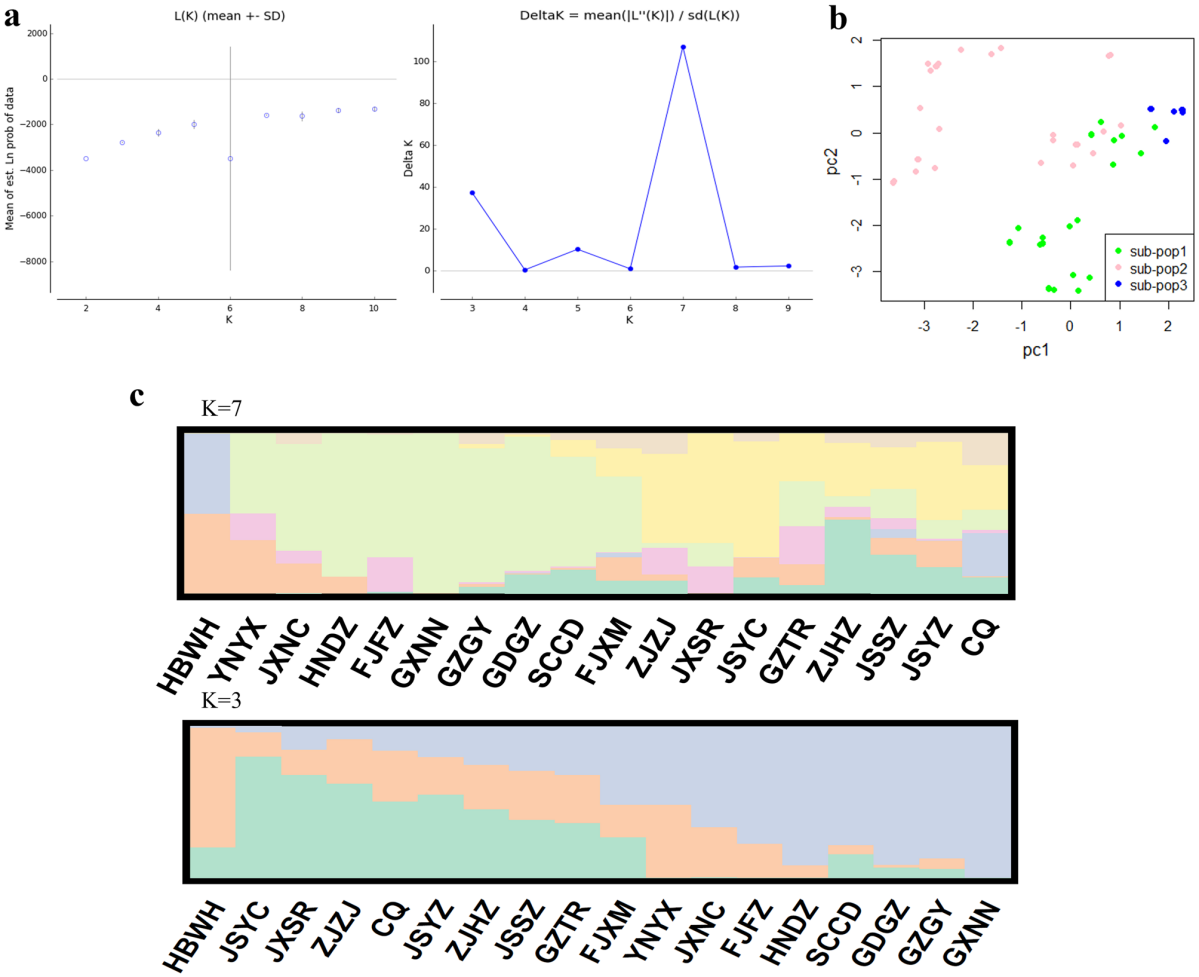
Three *P. canaliculata* subgroups in China based on rDNA data analysis. **(a)** Graph showing the number of inferred clusters(K) using Evanno’s delta(K) method. **(b)** Principal components analysis plot in the populations of 108 accessions, two-dimensional scales were used to reveal population stratification. **(c)** Population structure derived from STRUCTURE software. Each parallel row represents one individual and each colored segment in each column represents the percentage of the individual in the population. Abbreviations of the populations are described in Table S1 online.

From the rDNA data analyses, a Bayesian cluster analysis using STRUCTURE revealed an optimal value of K = 7 was the best fit for the 18 populations in China (Fig. 5a), and a relatively high Evanno’s delta (K) of K = 3 (Fig. 5a). Our TASSEL analyses also confirmed these three subgroups (Fig. S1b online). In the PCA, the populations from the second subgroup were located at the tips and connected the first and third subgroups (Fig. 5b). The F_ST_ value among all the geographic populations was 0.13573(Table 3), occurring primarily within populations, with the genetic variation within populations being caused by differences in the haplotypes among them. There was less genetic differentiation and more gene flow among different geographic populations (Fig. 4b), suggesting sufficient genetic exchange but some genetic differentiation between populations. The Exact test showed that 77.22% of the populations (Table S6 online) supported the random mating group hypothesis (*P* > 0.05), suggesting that the majority of the populations were not reproductively segregated from one another, and, therefore, there may be no cryptic *P. canaliculata* species found based on the *ITS1* gene alone. Three more subgroups were identified (Fig. 5c): the first subgroup included the FJXM, FJFZ, JXNC, HNDZ, SCCD, GDGZ, GZGY, and GXNN populations; the second subgroup contained the JXSR, ZJZJ, ZJHZ, JSYC, JSSZ, JSYZ, GZTR, and CQ populations; and the third subgroup contained the HBWH and YNYX populations.

### Demography history

**Figure 6:**
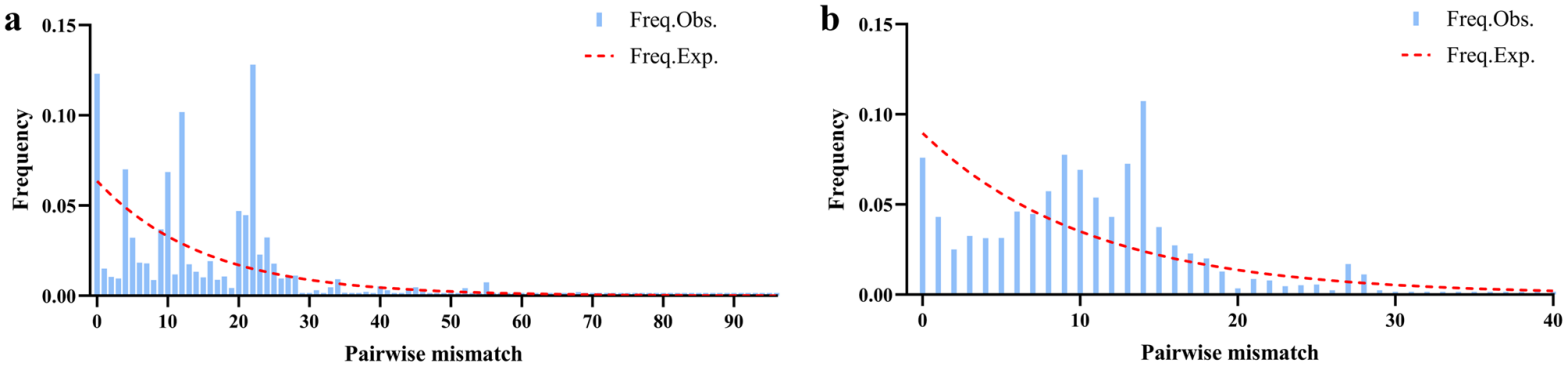
Mismatch distribution of *P. canaliculata*. **(a)** based on mtDNA *COI* gene. **(b)** based on rDNA *ITS1* gene.

To uncover the demography history of *P. canaliculata* in China, neutrality tests were conducted using Tajima’s D and Fu’s Fs statistics. Based on the mtDNA *COI* gene, the values of Tajima’s D for all the samples were positive (D = 0.12648), and Fu’s Fs statistic was positive (Fs = 3.37497) but not significant (*P* > 0.05). Tajima’s D and Fu’s Fs statistics for the FJFZ, GZGY, GZTR, HBWH, and ZJZJ populations were 0, indicating that these 5 populations conformed to a neutral evolution model. Meanwhile, Tajima’s D and Fu’s Fs statistics for the GDGZ, JSSZ, JSYZ, YNYX, and ZJHZ populations were positive but not significant(P > 0.05), which suggested that these 6 populations may have been in equilibrium or contraction (Table 4). Based on the rDNA *ITS1* gene, the values of Tajima’s D for all the samples were positive (D = -0.02777), while Fu’s Fs statistic was negative (Fs = 0.76997) but not significant (*P* > 0.05). Tajima’s D and Fu’s Fs statistics for the GXNN population were 0, indicating that conformed to a neutral evolution model. Instead, Tajima’s D and Fu’s Fs statistics for the GDGZ, JSYC, JXSR, and CQ populations were negative but not significant (*P* > 0.05), suggesting that these four populations may have been in an expansionary phase. Finally, Tajima’s D and Fu’s Fs statistics for the FJFZ, FJXM, GZTR, HNDZ, JXNC, and ZJHZ populations were positive but not significant (*P* > 0.05), indicating that these 6 populations may have been in equilibrium or contraction (Table 4). Based on our analysis, the multimodal mismatch distribution of all *P. canaliculata* samples (Fig. 6) may indicate that the *P. canaliculata* populations in China fit a neutral evolution model or that our samples covered several divergent populations.

**Table 4:** Neutraility test based on mtDNA and rDNA.

Population	mtDNA *COI*		rDNA *ITS1*	
	Tajima’s D	Fu’s fs	Tajima’s D	Fu’s fs
FJFZ	0	0	1.0337	3.02403
FJXM	0.84673	−0.42755	0.7583	0.70337
GDGZ	1.32483	6.35662	−1.22175	−0.65809
GXNN	−1.51833**	8.5007	0	0
GZGY	0	0	−1.42284*	4.00733
GZTR	0	0	0.58466	0.9038
HBWH	0	0	−1.29503	0.2969
HNDZ	−1.45122*	5.75519	2.14914	3.53529
JSSZ	2.14914	6.14109	−1.22175	2.54789
JSYC	−1.33698	2.59263	−0.29187	−0.62256
JSYZ	2.22223	6.1334	0.13811	−1.05269
JXNC	1.32483	6.35662	0.90194	1.69856
JXSR	−1.14991	1.37636	−0.28815	−0.83116
SCCD	−0.69622	0.41189	−0.77134	0.001
YNYX	0.37838	1.19214	−0.25987	0.25573
ZJHZ	1.37681	9.63012	0.93497	0.46112
ZJZJ	0	0	−0.22144	0.0554
CQ	−1.19369	2.36029	−0.00661	−0.46653

Significance thresholds: *0.01 < *P* < 0.05; **0.001 < *p* < 0.01.

## Discussion

For the *COI* gene, the genetic distance between groups of different geographical populations was within 7.0%, and the results of the molecular variance analysis (AMOVA) showed that the Fst value was 0.45296 (*P* = 0.0000), the Fst statistic at the population level showed a degree of genetic differentiation among the 18 populations, that is, values greater than 0.05 (*P* < 0.05) and 0.25 (*P* < 0.05) between 71.24 and 64.71% of the populations, respectively, and less than 0.05 between 13.73% of the populations, but the difference was not significant (*P* > 0.05). Previously, Balloux and Lugon-Moulin^49^ established that Fst values greater than 0.05 indicate the presence of genetic differentiation, with larger values indicating greater genetic differentiation. As a result, the values observed in our study indicated that a very great degree of genetic differentiation had occurred among the different geographical populations, with FJFZ and GXNN populations having the largest Fst value, followed by ZJHZ, ZJZJ, GZTR, and CQ populations. And FJFZ and GXNN populations shared one haplotype, GZTR shared one haplotype with JSYC and JSYZ, ZJHZ shared one haplotype with JSSZ, meanwhile ZJZJ was in the largest haplotype with CQ. In the gene flow results, we found that YNYX had an infinite gene flow with GDGZ, JSYZ, JXNC, and SCCD populations. According to the literature, the *Pomacea* spp. has been introduced into Yunnan Province multiple times. It was first introduced in 1985 into Dehong Dai and the Jingpo Autonomous Prefecture from the Aquatic Research Institute of Guangzhouand, in the same year, into the Xishuangbanna Dai Autonomous Prefecture as a farmer enrichment project. In 1986, it was brought into Jiangyin Farm, Huangping town, Heqing County^50^. In 1987, it was introduced into Xiashankou, Yousou town, Eryuan County, Dali Prefecture, subsequently abandoned in the Xihu and Donghu Wetlands and the farmland ditches around Xiashankou^51^, and brought back into the Dehong Prefecture during a study tour in 1990 to rear two snails, subsequently entered the farmland of Cangyuan County. In 2000, the infestation was heavier in the areas around Lake Erhai^52^, whose large-scale invasion began in 2010^53,54^, consistent with our results.

For the *ITS1* gene, the genetic distance between groups of different geographical populations was within 2.6%, and the results of the molecular variance analysis (AMOVA) showed the Fst value was 0.13573 (*P* = 0.0000), the Fst values between 24.18 and 17.65% of the populations were greater than 0.05 (*P* < 0.05) and 0.25 (*P* < 0.05), respectively, which produced a large genetic differentiation between GXNN and GZGY and other geographic populations, while Fst values was 0 for the GXNN and GZGY populations. According to Li et al.^55^, the first appearance of the *Pomacea* spp. in Guizhou Province corresponded to the introduction of fish fry from Guangxi into reservoirs and ponds in Pingtang County in the early 1990s, then spreading to rivers, ditches, and farmlands, while the earliest discovery of these species in Guangxi was in the mid-1980s in Fangchenggang city, and with uncertain origins. Therefore, it can be determined that GXNN and GZGY belong to the same invasion pathway and Fst values of GZGY with JXNC, HNDZ were less than 0.05 with Fst values less than 0 for JXNC with FJFZ, FJXM, GDGZ, SCCD, and HNDZ, therefore, JXNC, FJFZ, FJXM, GDGZ, GXNN, HNDZ, and GZGY were classified into a subgroup. The genetic differentiation between JXNC and JXSR populations was small (Fst = 0.1), and the gene flow large (Nm = 4.5). In contrast, there was greater genetic differentiation between GZGY and GZTR populations (Fst = 0.36667), suggesting different invasion pathways for different geographical populations in Guizhou Province. The Fst values of GZTR and JSSZ, JSYC, and JSYZ in Jiangsu Province were all less than 0.05, and the first discovery of *Pomacea* spp. in Jiangsu Province was in Zhangpu Town, Kunshan City^56^, however, the geographical distance between the two places is 1457 km, with no way to achieving population migration by relying on the free dispersal of snails, meaning that there must have been some influence from anthropogenic activities. The results of this study also found that there may have been population expansion in GDGZ, JSYC, JXSR, and CQ, preserving the genetic diversity of *P. canaliculata* and increasing the possibility of colonization in new environments, and that population dispersal occurred within China.

To sum up, the results obtained based on *COI* and *ITS1* have both similarities and differences due to the varied information they carry. The greatest genetic differentiation occurred in the GXNN population, which was first introduced into Guangdong Province, in mainland China, in 1981^57^, and then into Guangxi and Hainan Provinces from Guangdong Province in the mid-1980s^58,59^, autonomously spreading to Fujian Province in 1984^60^ In the same year, snails were introduced into Sichuan Province, and in 1987, 1988, and 2004, more specimens were introduced from different areas. In different time periods, snails were introduced from different areas into Yunnan Province^61,62^. At the end of the 1980s, after the introduction of overseas *Pomacea* specimens into Jiangxi Province due to a flood, the species spread to the whole province^63^. During the same period, specimens appeared in Ningbo, in Zhejiang Province, and again in the early 1990s, two introductions which we infer originated from different places^64,65^. In 1989 it was also introduced in Chongqing and gradually spread^66^, and in the early 1990s snails were introduced from Guangxi Province into Guizhou Province, where a genetically distinct geographic population (GZTR) also exists^55,67^. In 1998 it was introduced into Hubei Province, reintroduced in 2014, and spread by flooding in 2016^68,69^, and artificially introduced by GZTR into Jiangsu Province in 2003^56^. From the time of invasion and our results, we obtained that the GXNN, HNDZ, and FJ populations were introduced from Guangzhou; subsequently, SCCD, YNYX, JX, ZJ, and CQ populations were introduced from different regions, and through environmental influences and active dispersal, several populations at different times underwent sufficient genetic exchange.

The results obtained from different genes were analyzed, and it was found that the evolutionary rate based on *COI* gene was faster and carried more information; the Fst was greater than that obtained by *ITS1*; and the number of mutation sites (V = 232 and V = 89, respectively) single mutation sites (S = 118 and S = 33, respectively) was larger. A total of 30 haplotypes were based on *COI*, while 61 were based on *ITS1*. Although the haplotype diversity of *COI* gene (Hd = 0.877, *P* < 0.05) was less than that of *ITS1* gene (Hd = 0.924, *P* < 0.05), the nucleotide diversity of *COI* (Pi = 0.04304, *P* < 0.01) was greater than that of *ITS1* gene (Pi = 0.02336, *P* < 0.01). The Exact tests for the COI gene revealed the possible existence of a cryptic *P. canaliculata* species, which is consistent with the result of Lv et al.^70^ and Yang et al.^71^, who named this cryptic species as *Pomacea occulta*^72^, whereas Exact tests for *ITS1* gene did not support its existence. Further indicating that the information carried by the two genes is different and that mitochondrial DNA evolves at a faster rate.

Overall, *COI* can better reflect the origin, migration, and dispersal of a species in a certain geographic region and be used for the study of population history and dynamics, genealogical geography, genetic diversity, and phylogenetic classification, among other topics. More than 40 years have passed since the introduction of the *Pomacea* spp. to mainland China, and its genetic richness should be relatively evenly represented in its various populations rather than being concentrated in one or a few specific populations, which is in line with the following findings that the genetic diversity of *P. canaliculata* is relatively high in the populations in the lower reaches of the Yangtze River in China^32^, in Zhejiang Province^27^, in Suzhou City^28^, and in Nanchong City^29^ populations, all of the *P. canaliculata* populations showed high genetic diversity, and the Chinese population of *P. canaliculata* was closer to the Argentinean population^71^, and multiple invasions occurred in the different populations, which could obtain more genotypes from the original gene pool, thus facilitating the *P. canaliculata* population adaptation and expansion^73^. We hypothesized that the evolutionary history the *Pomacea* spp. in China may have also been complexly influenced by some abiotic (e.g., climate) and biotic components (e.g., host plants). We need more studies, both theoretical and experimental, to reach a full understanding of evolution and variation at the molecular level of this specie.

### Supplementary Information

Supplementary Information.

## Data Availability

The data that support the findings of this study are openly available in NCBI: GenBank accession nos. PP461874-PP461981 and GenBank accession nos. PP448193-PP448300.
