# Supplementary material for: Population genetic structure of Pomacea canaliculata in China based on the COI and ITS1 genes
Source: Sci Rep. 2024 May 27;14:12045. doi: 10.1038/s41598-024-62554-6 (PMC11130151; doi:10.1038/s41598-024-62554-6)
Supplement: Supplementary file 1 — Supplementary Information. [file 41598_2024_62554_MOESM1_ESM.docx]

Population genetic structure of *Pomacea.canaliculata* in China based on the *COI* and *ITS1* genes

Ran Wei^1,2^, Ya-Wen Chang^1^, Hong-Fang Xie^3^, Cheng-dong Wu^4^, Deng-Rong Yuan^3^, Wei-Rong Gong^5^, Yu-Zhou Du^1,*^

^1^ College of plant protection, Yangzhou University, Yangzhou 225009, China

^2^ College of bioscience and biotechnology, Yangzhou University, Yangzhou 225009, China

^3^ Plant Protection and Quarantine Station of Nanjing City, Jiangsu Province, Nanjing 210029, China

^4^ Pukou Agricultural Technology Extension Center of Nanjing City, Nanjing, 211800, China

^5^ Plant Protection and Quarantine Station of Jiangsu Province, Nanjing 210036, China

^*^ Corresponding author, E-mail: yzdu@yzu.edu.cn, Tel: 13952751570

**Table S1 Distribution of mitochondrial DNA *COI* haplotype**

| Haplotypes | Numbers of individuals | *P. canaliculata* individuals and populations |
| --- | --- | --- |
| Hap1 | 1 | CQ_1 |
| Hap2 | 22 | CQ_2 CQ_3 CQ_4 CQ_6 GDGZ_2 GDGZ_5 JSYZ_4 JSYZ_5 JSYZ_6 JXNC_1 JXNC_2 JXNC_4 JXNC_5 SCCD_2 YNYX_1 YNYX_3 ZJZJ_1 ZJZJ_2 ZJZJ_3 ZJZJ_4 ZJZJ_5 ZJZJ_6 |
| Hap3 | 1 | CQ_5 |
| Hap4 | 11 | FJFZ-1 FJFZ-2 FJFZ-3 FJFZ-4 FJFZ-5 FJFZ-6 GXNN_1 GXNN_3 GXNN_4 GXNN_5 GXNN_6 |
| Hap5 | 1 | FJXM_1 |
| Hap6 | 1 | FJXM_2 |
| Hap7 | 1 | FJXM_3 |
| Hap8 | 2 | FJXM_4 JXSR_2 |
| Hap9 | 25 | FJXM_5 GDGZ_1 GDGZ_3 GDGZ_4 GDGZ_6 GZGY_1 GZGY_2 GZGY_3 GZGY_4 GZGY_5 GZGY_6 HBWH_1 HBWH_2 HBWH_3 HBWH_4 HBWH_5 HBWH_6 GNDZ_1 GNDZ_2 GNDZ_3 GNDZ_6 JXNC_3 JXNC_6 JXSR_1 JXSR_3 |
| Hap10 | 1 | FJXM_6 |
| Hap11 | 1 | GXNN_2 |
| Hap12 | 15 | GZTR_1 GZTR_2 GZTR_3 GZTR_4 GZTR_5 GZTR_6 JSYC_1 JSYC_3 JSYC_4 JSYC_5 JSYC_6 JSYZ_1 JSYZ_2 JSYZ_3 SCCD_1 |
| Hap13 | 1 | HNDZ_4 |
| Hap14 | 1 | HNDZ_5 |
| Hap15 | 3 | JSSZ_1 JSSZ_3 JSSZ_4 |
| Hap16 | 1 | JSSZ_2 |
| Hap17 | 6 | JSSZ_5 JSSZ_6 ZJHZ_1 ZJHZ_2 ZJHZ_4 ZJHZ_5 |
| Hap18 | 1 | JSYC_2 |
| Hap19 | 1 | JXSR_4 |
| Hap20 | 1 | JXSR_5 |
| Hap21 | 1 | JXSR_6 |
| Hap22 | 1 | SCCD_3 |
| Hap23 | 1 | SCCD_4 |
| Hap24 | 1 | SCCD_5 |
| Hap25 | 1 | SCCD_6 |
| Hap26 | 1 | YNYX_2 |
| Hap27 | 1 | YNYX_4 |
| Hap28 | 1 | YNYX_5 |
| Hap29 | 1 | YNYX_6 |
| Hap30 | 2 | ZJHZ_3 ZJHZ_6 |

**Table S2 Distribution of ribosomal DNA *ITS1* haplotype**

| Haplotypes | Numbers of individuals | *P. canaliculata* individuals and populations |
| --- | --- | --- |
| Hap1 | 28 | FJFZ_1 FJFZ_4 FJFZ_5 FJXM_1 FJXM_3 GDGZ_2 GDGZ_3 GXNN_1 GXNN_2 GXNN_3 GXNN_4 GXNN_5 GXNN_6 GZGY_1 GZGY_3 GZGY_4 GZGY_5 GZGY_6 HNDZ_1 HNDZ_3 HNDZ_4 HNDZ_5 JXNC_3 JXNC_4 JXNC_5 SCCD_2 SCCD_6 YNYX_1 |
| Hap2 | 3 | FJFZ_2 FJFZ_3 ZJZJ_1 |
| Hap3 | 1 | FJFZ_6 |
| Hap4 | 1 | FJXM_2 |
| Hap5 | 1 | FJXM_4 |
| Hap6 | 1 | FJXM_5 |
| Hap7 | 10 | FJXM_6 GZTR_5 GZTR_6 JSYC_2 JSYC_4 JSYC_5 JSYC_6 JSYZ_5 ZJHZ_3 ZJHZ_4 |
| Hap8 | 1 | GDGZ_1 |
| Hap9 | 1 | GDGZ_4 |
| Hap10 | 1 | GDGZ_5 |
| Hap11 | 1 | GDGZ_6 |
| Hap12 | 1 | GZGY_2 |
| Hap13 | 1 | GZTR_1 |
| Hap14 | 1 | GZTR_2 |
| Hap15 | 1 | GZTR_3 |
| Hap16 | 1 | GZTR_4 |
| Hap17 | 1 | HNDZ_2 |
| Hap18 | 1 | HNDZ_6 |
| Hap19 | 3 | HBWH_1 HBWH_2 CQ_3 |
| Hap20 | 1 | HBWH_3 |
| Hap21 | 2 | HBWH_4 HBWH_6 |
| Hap22 | 1 | HBWH_5 |
| Hap23 | 1 | JSYC_1 |
| Hap24 | 1 | JSYC_3 |
| Hap25 | 1 | JSSZ_1 |
| Hap26 | 1 | JSSZ_2 |
| Hap27 | 1 | JSSZ_3 |
| Hap28 | 2 | JSSZ_4 ZJHZ_1 |
| Hap29 | 1 | JSSZ_5 |
| Hap30 | 4 | JSSZ_6 ZJZJ_2 ZJZJ_5 CQ_5 |
| Hap31 | 2 | JSYZ_1 JXSR_3 |
| Hap32 | 1 | JSYZ_2 |
| Hap33 | 1 | JSYZ_3 |
| Hap34 | 1 | JSYZ_4 |
| Hap35 | 1 | JSYZ_6 |
| Hap36 | 1 | JXNC_1 |
| Hap37 | 1 | JXNC_2 |
| Hap38 | 1 | JXNC_6 |
| Hap39 | 1 | JXSR_1 |
| Hap40 | 1 | JXSR_2 |
| Hap41 | 1 | JXSR_4 |
| Hap42 | 1 | JXSR_5 |
| Hap43 | 1 | JXSR_6 |
| Hap44 | 1 | SCCD_1 |
| Hap45 | 1 | SCCD_3 |
| Hap46 | 1 | SCCD_4 |
| Hap47 | 1 | SCCD_5 |
| Hap48 | 2 | YNYX_2 YNYX_3 |
| Hap49 | 1 | YNYX_4 |
| Hap50 | 1 | YNYX_5 |
| Hap51 | 1 | YNYX_6 |
| Hap52 | 1 | ZJHZ_2 |
| Hap53 | 1 | ZJHZ_5 |
| Hap54 | 1 | ZJHZ_6 |
| Hap55 | 1 | ZJZJ_3 |
| Hap56 | 1 | ZJZJ_4 |
| Hap57 | 1 | ZJZJ_6 |
| Hap58 | 1 | CQ_1 |
| Hap59 | 1 | CQ_2 |
| Hap60 | 1 | CQ_4 |
| Hap61 | 1 | CQ_6 |

**Table S3 Sequence base transitions, number of inversions and frequency of base pairs at different loci in *COI* gene fragments from different geographical populations**

| Domain | ii | si | sv | R | location | | | | | | | | | | | | | | | | |
| --- | --- | --- | --- | --- | --- | --- | --- | --- | --- | --- | --- | --- | --- | --- | --- | --- | --- | --- | --- | --- | --- |
|  |  |  |  |  | TT | TC | TA | TG | CT | CC | CA | CG | AT | AC | AA | AG | GT | GC | GA | GG | Total |
| Avg | 410 | 17 | 8 | 2 | 177 | 3 | 2 | 1 | 3 | 62 | 1 | 1 | 1 | 1 | 100 | 5 | 1 | 1 | 6 | 71 | 435.3 |
| 1st | 140 | 4 | 2 | 1.4 | 59 | 1 | 0 | 0 | 1 | 24 | 0 | 0 | 0 | 0 | 36 | 1 | 0 | 0 | 1 | 21 | 145.7 |
| 2nd | 136 | 6 | 3 | 1.9 | 66 | 1 | 0 | 1 | 1 | 21 | 0 | 0 | 0 | 0 | 28 | 2 | 1 | 0 | 2 | 22 | 144.9 |
| 3rd | 135 | 7 | 3 | 2.8 | 52 | 1 | 1 | 0 | 1 | 18 | 0 | 0 | 1 | 0 | 37 | 2 | 0 | 0 | 3 | 28 | 144.7 |

**Table S4 Sequence base transitions, number of inversions and frequency of base pairs at different loci in *ITS1* gene fragments from different geographical populations**

| Domain | location | | | | | | | | | | | | | | | | | | | | |
| --- | --- | --- | --- | --- | --- | --- | --- | --- | --- | --- | --- | --- | --- | --- | --- | --- | --- | --- | --- | --- | --- |
|  | ii | si | sv | R | TT | TC | TA | TG | CT | CC | CA | CG | AT | AC | AA | AG | GT | GC | GA | GG | Total |
| Avg | 436 | 6 | 8 | 0.7 | 100 | 1 | 2 | 1 | 1 | 110 | 1 | 0 | 1 | 1 | 98 | 2 | 1 | 1 | 1 | 129 | 450.3 |
| 1st | 146 | 1 | 3 | 0.4 | 26 | 0 | 1 | 1 | 0 | 45 | 0 | 0 | 1 | 0 | 30 | 0 | 0 | 0 | 0 | 46 | 150.2 |
| 2nd | 144 | 2 | 2 | 0.9 | 37 | 0 | 0 | 0 | 0 | 38 | 1 | 0 | 0 | 1 | 37 | 1 | 0 | 0 | 1 | 32 | 148.5 |
| 3rd | 147 | 2 | 2 | 1 | 37 | 0 | 1 | 0 | 1 | 27 | 0 | 0 | 1 | 0 | 32 | 1 | 0 | 0 | 1 | 51 | 151.5 |

**Table S5 Exact test based mtDNA *COI***

|  | FJFZ | FJXM | GDGZ | GXNN | GZGY | GZTR | HBWH | HNDZ | JSSZ | JSYC | JSYZ | JXNC | JXSR | SCCD | YNYX | ZJHZ | ZJZJ | CQ |
| --- | --- | --- | --- | --- | --- | --- | --- | --- | --- | --- | --- | --- | --- | --- | --- | --- | --- | --- |
| FJFZ |  |  |  |  |  |  |  |  |  |  |  |  |  |  |  |  |  |  |
| FJXM | 0.00197+-0.0004 |  |  |  |  |  |  |  |  |  |  |  |  |  |  |  |  |  |
| GDGZ | 0.00188+-0.0004 | 0.13364+-0.0042 |  |  |  |  |  |  |  |  |  |  |  |  |  |  |  |  |
| GXNN | 1.00000+-0.0000 | 0.01461+-0.0018 | 0.00233+-0.0005 |  |  |  |  |  |  |  |  |  |  |  |  |  |  |  |
| GZGY | 0.00234+-0.0002 | 0.01598+-0.0013 | 0.45730+-0.0011 | 0.00225+-0.0005 |  |  |  |  |  |  |  |  |  |  |  |  |  |  |
| GZTR | 0.00257+-0.0002 | 0.00214+-0.0005 | 0.00287+-0.0004 | 0.00220+-0.0004 | 0.00199+-0.0002 |  |  |  |  |  |  |  |  |  |  |  |  |  |
| HBWH | 0.00215+-0.0003 | 0.01448+-0.0015 | 0.45258+-0.0011 | 0.00150+-0.0002 | -2 | 0.00245+-0.0003 |  |  |  |  |  |  |  |  |  |  |  |  |
| HNDZ | 0.00238+-0.0004 | 0.24168+-0.0052 | 0.45741+-0.0041 | 0.00543+-0.0009 | 0.45285+-0.0025 | 0.00289+-0.0005 | 0.45758+-0.0026 |  |  |  |  |  |  |  |  |  |  |  |
| JSSZ | 0.00237+-0.0005 | 0.09156+-0.0046 | 0.00436+-0.0007 | 0.00470+-0.0008 | 0.00191+-0.0004 | 0.00195+-0.0003 | 0.00266+-0.0003 | 0.00812+-0.0008 |  |  |  |  |  |  |  |  |  |  |
| JSYC | 0.00233+-0.0004 | 0.01643+-0.0013 | 0.00244+-0.0005 | 0.00440+-0.0006 | 0.00208+-0.0003 | 1.00000+-0.0000 | 0.00207+-0.0004 | 0.00564+-0.0006 | 0.00493+-0.0006 |  |  |  |  |  |  |  |  |  |
| JSYZ | 0.00214+-0.0003 | 0.05007+-0.0020 | 0.03604+-0.0007 | 0.00211+-0.0004 | 0.00146+-0.0003 | 0.18194+-0.0015 | 0.00204+-0.0002 | 0.00219+-0.0004 | 0.00640+-0.0008 | 0.18383+-0.0021 |  |  |  |  |  |  |  |  |
| JXNC | 0.00178+-0.0004 | 0.05473+-0.0032 | 0.56537+-0.0016 | 0.00221+-0.0004 | 0.05962+-0.0013 | 0.00206+-0.0003 | 0.06160+-0.0010 | 0.05964+-0.0016 | 0.00328+-0.0004 | 0.00223+-0.0005 | 0.09093+-0.0011 |  |  |  |  |  |  |  |
| JXSR | 0.00301+-0.0004 | 1.00000+-0.0000 | 0.30526+-0.0054 | 0.00894+-0.0012 | 0.06316+-0.0021 | 0.00190+-0.0005 | 0.06157+-0.0010 | 0.55755+-0.0037 | 0.04333+-0.0026 | 0.01012+-0.0013 | 0.01926+-0.0010 | 0.11267+-0.0032 |  |  |  |  |  |  |
| SCCD | 0.00191+-0.0006 | 1.00000+-0.0000 | 0.06534+-0.0027 | 0.01674+-0.0012 | 0.00152+-0.0003 | 0.01566+-0.0009 | 0.00219+-0.0004 | 0.06343+-0.0050 | 0.09407+-0.0036 | 0.08081+-0.0038 | 0.35729+-0.0045 | 0.13630+-0.0080 | 0.45140+-0.0055 |  |  |  |  |  |
| YNYX | 0.00143+-0.0005 | 0.45117+-0.0096 | 0.10675+-0.0045 | 0.01252+-0.0015 | 0.00236+-0.0006 | 0.00281+-0.0005 | 0.00273+-0.0008 | 0.04000+-0.0037 | 0.04551+-0.0015 | 0.00848+-0.0010 | 0.18494+-0.0045 | 0.31481+-0.0055 | 0.21005+-0.0050 | 1.00000+-0.0000 |  |  |  |  |
| ZJHZ | 0.00228+-0.0004 | 0.03372+-0.0024 | 0.00440+-0.0005 | 0.00169+-0.0002 | 0.00206+-0.0005 | 0.00227+-0.0004 | 0.00213+-0.0002 | 0.00380+-0.0005 | 0.06910+-0.0020 | 0.00178+-0.0005 | 0.00215+-0.0002 | 0.00486+-0.0006 | 0.01612+-0.0012 | 0.03541+-0.0034 | 0.01589+-0.0014 |  |  |  |
| ZJZJ | 0.00232+-0.0002 | 0.00257+-0.0007 | 0.06037+-0.0013 | 0.00249+-0.0003 | 0.00190+-0.0002 | 0.00176+-0.0003 | 0.00223+-0.0003 | 0.00169+-0.0003 | 0.00221+-0.0004 | 0.00203+-0.0003 | 0.18005+-0.0023 | 0.45304+-0.0022 | 0.00182+-0.0005 | 0.01560+-0.0011 | 0.06154+-0.0032 | 0.00144+-0.0003 |  |  |
| CQ | 0.00293+-0.0006 | 0.05982+-0.0031 | 0.06265+-0.0016 | 0.00616+-0.0006 | 0.00212+-0.0004 | 0.00225+-0.0002 | 0.00130+-0.0002 | 0.01159+-0.0018 | 0.00785+-0.0011 | 0.00643+-0.0011 | 0.18104+-0.0047 | 0.45398+-0.0047 | 0.03242+-0.0016 | 0.25481+-0.0029 | 0.56433+-0.0054 | 0.00380+-0.0004 | 0.45458+-0.0016 |  |

**Table S6 Exact test based rDNA *ITS1***

|  | FJFZ | FJXM | GDGZ | GXNN | GZGY | GZTR | HBWH | HNDZ | JSSZ | JSYC | JSYZ | JXNC | JXSR | SCCD | YNYX | ZJHZ | ZJZJ | CQ |
| --- | --- | --- | --- | --- | --- | --- | --- | --- | --- | --- | --- | --- | --- | --- | --- | --- | --- | --- |
| FJFZ |  |  |  |  |  |  |  |  |  |  |  |  |  |  |  |  |  |  |
| FJXM | 0.56407+-0.0055 |  |  |  |  |  |  |  |  |  |  |  |  |  |  |  |  |  |
| GDGZ | 0.57470+-0.0052 | 1.00000+-0.0000 |  |  |  |  |  |  |  |  |  |  |  |  |  |  |  |  |
| GXNN | 0.17962+-0.0025 | 0.06385+-0.0022 | 0.06323+-0.0024 |  |  |  |  |  |  |  |  |  |  |  |  |  |  |  |
| GZGY | 0.30423+-0.0051 | 0.24311+-0.0056 | 0.24792+-0.0038 | 1.00000+-0.0000 |  |  |  |  |  |  |  |  |  |  |  |  |  |  |
| GZTR | 0.04198+-0.0024 | 0.53922+-0.0060 | 0.22040+-0.0059 | 0.00176+-0.0004 | 0.01044+-0.0014 |  |  |  |  |  |  |  |  |  |  |  |  |  |
| HBWH | 0.45351+-0.0037 | 0.56832+-0.0057 | 0.57075+-0.0056 | 0.45298+-0.0033 | 1.00000+-0.0000 | 0.03282+-0.0024 |  |  |  |  |  |  |  |  |  |  |  |  |
| HNDZ | 0.02285+-0.0019 | 0.09874+-0.0034 | 0.09871+-0.0023 | 0.00280+-0.0003 | 0.00705+-0.0010 | 0.10084+-0.0026 | 0.01826+-0.0012 |  |  |  |  |  |  |  |  |  |  |  |
| JSSZ | 0.00837+-0.0007 | 0.12903+-0.0031 | 0.03670+-0.0016 | 0.00267+-0.0005 | 0.00678+-0.0011 | 0.55462+-0.0050 | 0.01321+-0.0015 | 0.01680+-0.0014 |  |  |  |  |  |  |  |  |  |  |
| JSYC | 0.09720+-0.0039 | 0.45674+-0.0081 | 0.45747+-0.0064 | 0.00211+-0.0005 | 0.01426+-0.0016 | 0.45128+-0.0061 | 0.05984+-0.0041 | 0.21545+-0.0054 | 0.06021+-0.0036 |  |  |  |  |  |  |  |  |  |
| JSYZ | 0.09092+-0.0037 | 0.69789+-0.0031 | 0.45549+-0.0072 | 0.00248+-0.0007 | 0.01337+-0.0013 | 1.00000+-0.0000 | 0.05818+-0.0023 | 0.21873+-0.0044 | 0.24783+-0.0053 | 1.00000+-0.0000 |  |  |  |  |  |  |  |  |
| JXNC | 0.48926+-0.0046 | 1.00000+-0.0000 | 1.00000+-0.0000 | 0.18489+-0.0038 | 0.54007+-0.0037 | 0.09598+-0.0046 | 1.00000+-0.0000 | 0.04239+-0.0022 | 0.01872+-0.0010 | 0.18324+-0.0067 | 0.18147+-0.0071 |  |  |  |  |  |  |  |
| JXSR | 0.08261+-0.0043 | 0.44480+-0.0075 | 0.45238+-0.0064 | 0.00242+-0.0008 | 0.01659+-0.0009 | 0.46378+-0.0055 | 0.06673+-0.0023 | 0.21723+-0.0061 | 0.06180+-0.0030 | 1.00000+-0.0000 | 1.00000+-0.0000 | 0.18023+-0.0061 |  |  |  |  |  |  |
| SCCD | 0.56426+-0.0042 | 1.00000+-0.0000 | 1.00000+-0.0000 | 0.06078+-0.0019 | 0.24171+-0.0049 | 0.20779+-0.0050 | 0.55446+-0.0052 | 0.10268+-0.0019 | 0.03565+-0.0026 | 0.46268+-0.0068 | 0.45355+-0.0076 | 1.00000+-0.0000 | 0.45587+-0.0064 |  |  |  |  |  |
| YNYX | 0.16502+-0.0024 | 0.54800+-0.0041 | 0.54200+-0.0045 | 0.01494+-0.0008 | 0.05545+-0.0025 | 0.20964+-0.0044 | 0.13882+-0.0033 | 0.10101+-0.0025 | 0.03476+-0.0026 | 0.44099+-0.0049 | 0.45855+-0.0084 | 0.28300+-0.0045 | 0.44451+-0.0069 | 0.54719+-0.0071 |  |  |  |  |
| ZJHZ | 0.04088+-0.0029 | 0.53740+-0.0063 | 0.20951+-0.0048 | 0.00284+-0.0008 | 0.01084+-0.0010 | 1.00000+-0.0000 | 0.03444+-0.0022 | 0.10245+-0.0021 | 0.56369+-0.0069 | 0.69960+-0.0047 | 1.00000+-0.0000 | 0.09053+-0.0033 | 0.45581+-0.0042 | 0.20713+-0.0067 | 0.21276+-0.0067 |  |  |  |
| ZJZJ | 0.20223+-0.0035 | 0.21384+-0.0054 | 0.21022+-0.0067 | 0.00170+-0.0004 | 0.00951+-0.0013 | 0.20272+-0.0062 | 0.03334+-0.0016 | 0.10073+-0.0020 | 0.03537+-0.0018 | 1.00000+-0.0000 | 0.44737+-0.0083 | 0.09139+-0.0034 | 0.45870+-0.0055 | 0.21734+-0.0041 | 0.21482+-0.0052 | 0.20835+-0.0053 |  |  |
| CQ | 0.09138+-0.0047 | 0.44595+-0.0062 | 0.44857+-0.0074 | 0.00135+-0.0005 | 0.01372+-0.0008 | 0.45065+-0.0074 | 0.06193+-0.0031 | 0.55601+-0.0083 | 0.06254+-0.0038 | 1.00000+-0.0000 | 1.00000+-0.0000 | 0.18390+-0.0065 | 1.00000+-0.0000 | 0.45301+-0.0060 | 0.44870+-0.0065 | 0.45560+-0.0066 | 1.00000+-0.0000 |  |


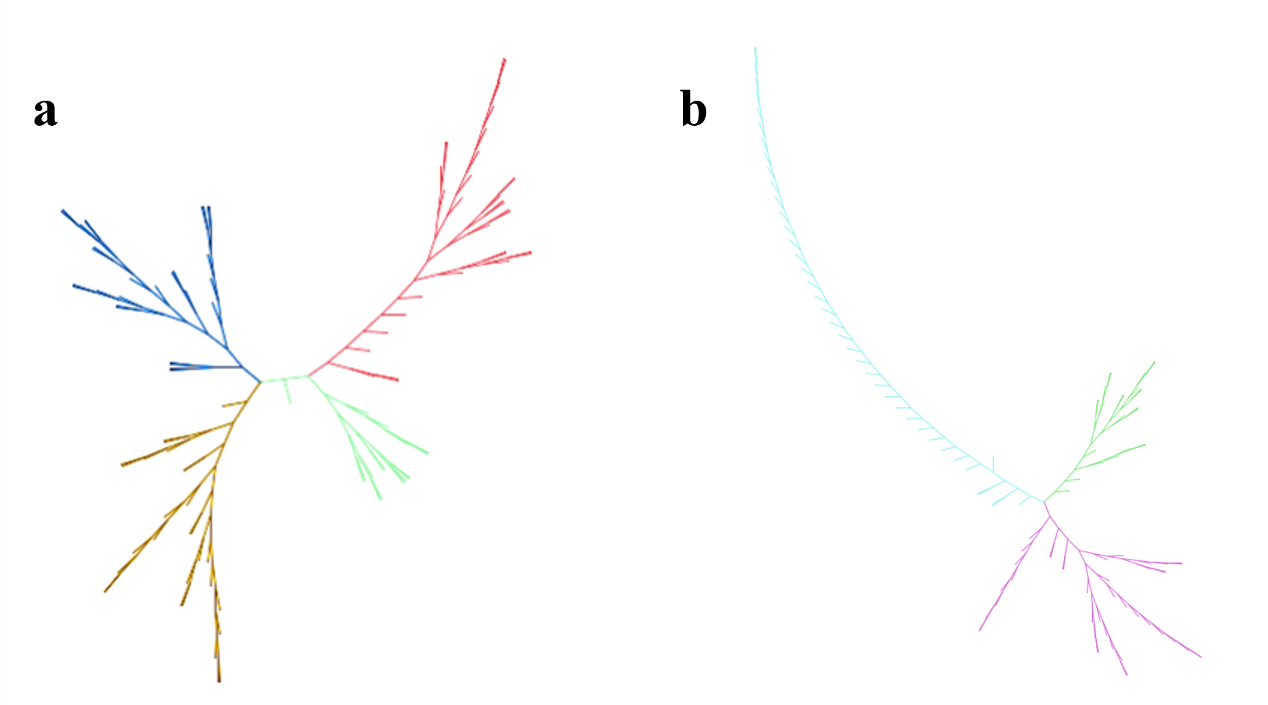


Fig S1 A neighbor-joining tree. **(a)** based on mtDNA *COI* gene. **(b)** based on rDNA *ITS1* gene.
